# Study on the cultivation of Lactobacillus using rice wine lees and protection capabilities during spray and freeze drying

**DOI:** 10.1016/j.fochx.2025.102741

**Published:** 2025-07-09

**Authors:** Liya Tian, Xing Wang, Qingyun Lyu, Lijie Zhu, Lei Chen, Xi Chen, Wenping Ding

**Affiliations:** a School of Food Science and Engineering, Wuhan Polytechnic University, Wuhan 430023, China; b Key Laboratory for Deep Processing of Major Grain and Oil, Wuhan Polytechnic University, Ministry of Education, Wuhan 430023, China

**Keywords:** Rice wine lees, Lactobacillus, Spray drying, Physicochemical properties

## Abstract

Rice wine lees (RWL), a byproduct of traditional Chinese rice wine production, can be used to cultivate *Lactobacillus* strains and enhance their viability during spray drying (28.5 % survival) and freeze drying (31.8 % survival). Spray-dried powders exhibited uniform spherical morphology, smaller particle size, and improved gastric tolerance, while freeze-dried samples retained greater odour diversity, as confirmed by GC-IMS analysis. Electronic nose analysis indicated reduced volatile compounds in spray-dried probiotics. Both drying methods produced powders stable at 4 °C and 20 °C for two months, maintaining viable counts above 10^7^ CFU/g. These findings highlight the potential of RWL to improve processing adaptability and storage stability of microbial formulations, offering an eco-friendly substrate for probiotic applications.

## Highlights

• Rice wine lees extract as a novel medium for Lactobacillus cultivation.
• Improved survival of Lactobacillus during drying through RWL extract centrifuged precipitate.
• Enhanced tolerance of probiotic powders to simulated gastric fluid.
• Excellent storage stability of probiotic powders at low temperatures, suggesting potential for commercial use.

## Graphical abstract

**Figure:**
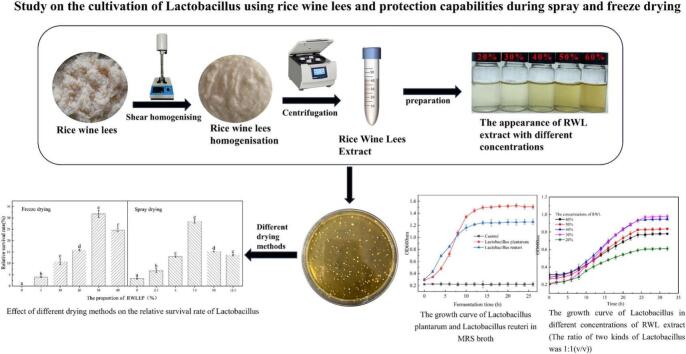

## 1 Introduction

Rice wine is brewed from glutinous rice and fermentation starter. It has a sweet taste, mellow flavor, and low alcohol content and is loved by the general public. It is rich in nutrients, containing sugars, proteins, amino acids, trace elements, etc. RWL is byproduct of rice wine fermentation. A large quantity of RWL is produced annually. It was estimated that in 2013, approximately 3.1 billion liters of rice wine and about 1 million metric tons of RWL were produced (Zhu et al., 2020). Currently, RWL are primarily used as animal feed, without full utilization. Owing to their richness in nutrients related to one‑carbon metabolism and high protein content (Rani et al., 2018). RWL has become a research focus in food applications. Functional components can be extracted from RWL, for example, antioxidant peptides (Wu et al., 2023) angiotensin-I converting enzyme inhibitory (ACEi) peptides (He et al., 2021) and rice protein (Zhao et al., 2024). Additionally, RWL are rich in proteins, carbohydrates, and amino acids, which are beneficial for microbial cultivation and growth. Such as producing a yeast culture using solid-state fermentation (Hu et al., 2014) or bio-converting RWL into high-value proteins through mixed yeast-fungus cultures (Zhu et al., 2020). Hong et al. employed fruit and vegetable juices as high-quality substrates for fermentation to cultivate probiotics (Hong et al., 2024). In contrast to conventional culture media, the preparation of RWL is remarkably straightforward, and RWL offers significant cost advantages as it is primarily used as animal feed and is more economically affordable. Although the cultivation efficiency of RWL is slightly lower than that of MRS medium, optimizing its culture conditions can still yield satisfactory microbial growth. Furthermore, RWL demonstrates practical benefits in industrial applications due to its simple preparation process, minimal equipment requirements, and operational convenience.

The *Lactobacillus* is commonly used as probiotic that can utilize carbohydrates for growth metabolism (Kalam et al., 2018). It is widely used in the food industry for various fermented products. Its metabolic byproducts play crucial roles in physiological functions such as regulating gut microbiota, enhancing immunity, and exerting antimicrobial and anticancer effects (Du et al., 2022). *Lactobacillus plantarum* and *Lactobacillus reuteri* are symbiotic microbes in humans commonly used as probiotics, beneficially impacting host health by preventing chronic diseases like inflammatory bowel diseases (Wang et al., 2021). The former, with high acid and bile salt resistance, colonizes the gut effectively, modulates the intestinal microbiota, and is widely used in food fermentation and probiotics (Huang et al., 2015). The latter produces unique antimicrobial substances to suppress harmful gut bacteria and maintain microbial balance (Mu et al., 2018). *Lactobacillus* is one of the dominant bacterial communities in the rice wine fermentation process (Ke et al., 2014). The *Lactobacillus* is particularly sensitive to the harsh conditions found in many foods and in the human gut (Yao et al., 2020). An adequate solution to improve probiotic survival during their processing is their microencapsulation (Semyonov et al., 2011). Polysaccharides and proteins can serve as excellent matrix materials for encapsulating probiotics. These compounds form a protective barrier around the probiotic cells (Mohamadzadeh et al., 2024).

In industrial processing, spray-drying and freeze-drying are the two most commonly employed drying techniques. These drying methods may impact microbial viability. Protective agents are therefore critical to mitigate such damage. Yang et al. optimized freeze-drying parameters and protective formulations, achieving a post-lyophilization viability of 89.8 % (Yang, 2015). Sui et al. further refined cryoprotectant screening and process adjustments, attaining a survival rate of 39.2 % (Sui et al., 2016). For spray-drying, Xiong et al. demonstrated that a protective mixture comprising 5 % skim milk, 5 % trehalose, and 3 % sodium glutamate significantly enhanced *Lactobacillus* survival to 89.95 % (Xiong & Huang, 2015). After extracting the RWL extract in this study, the resulting extract is primarily composed of high-molecular-weight starch and proteins, and can be used as a matrix material for the microencapsulation of lactic acid bacteria (Li et al., 2013). The study analyzed the basic components of RWL, prepared RWL extract, and used it for *Lactobacillus* cultivation and protectant during drying. Probiotic powders were produced via freeze-drying and spray-drying methods, and their gastrointestinal tolerance, microstructure, particle size, flavor compounds, and other physicochemical properties were evaluated. We hope that our results will broaden the way for the comprehensive utilization of RWL, solve the environmental pollution problem, and create greater economic benefits.

## 2 Materials and methods

### 2.1 Materials

RWL was obtained and collected from a local rice wine distillery (Macheng Donggucheng Liquor and Food Co., Ltd., Macheng, China) and stored at 4 °C for subsequent analysis. The freeze-dried powder of *Lactobacillus plantarum* ATCC8014 and *Lactobacillus reuteri* ATCC23272 were procured from Beijing Zhongke Quality Inspection Biotechnology Co., Ltd. (Beijing, China) and stored at −20 °C for further use. MRS broth and MRS agar culture medium were purchased from Shanghai Shenshi Biochemical Technology Co., Ltd. (Shanghai, China). R30388 simulated gastric fluid and R30384 artificial intestinal juice purchased from Shanghai Yuanye Bio-Technology Co., Ltd. (Shanghai, China). All other chemicals used in the experiment were analytical grade.

### 2.2 Preparation of samples

#### 2.2.1 Preparation of RWL extract

Ultrapure water was added to RWL to achieve mass ratios of 20 %, 30 %, 40 %, 50 %, and 60 %, respectively. The mixtures were homogenized using an L12-P126 high-speed homogenizer (Jiuyang Co., Ltd., Jinan, China) at 12,000 rpm for 5 min. Subsequently, the homogenized RWL suspensions were transferred to a 1000 mL beaker and subjected to ultrasonic treatment at 200 W for 15 min, followed by thermal extraction in a water bath at 75 °C for 30 min. After extraction, the samples were sterilized in a YXQ-50SII autoclave (Shanghai Boxun Medical Biological Instrument Co., Ltd., Shanghai, China) at 121 °C for 20 min. Following sterilization, the samples were centrifuged at 5000 rpm for 10 min, and the supernatant was collected as the RWL extract, which was used for *Lactobacillus* cultivation and growth curve analysis. The resulting precipitate (RWLEP) was stored at 4 °C and served as the matrix material for subsequent *Lactobacillus* microencapsulation in drying experiments.

#### 2.2.2 Activation of *Lactobacillus plantarum* and Lactobacillus reuteir

Following the method described by Liu et al. the lyophilized powder of *Lactobacillus plantarum* and *Lactobacillus reuteri* was reactivated in MRS broth at 37 °C for 24 h. The culture solution was transferred to MRS solid medium and incubated at 37 °C for 24 h (Liu et al., 2022). A single colony from the agar plate was aseptically inoculated into 50 mL of sterile MRS broth and incubated at 37 °C for 24 h. The resulting culture was used as the activated seed culture.

#### 2.2.3 Lactobacillus culture and growth curve

Following the method described by Liu et al. 2.50 mL of activated seed cultures of *Lactobacillus reuteri* and *Lactobacillus plantarum* were transferred into 100 mL MRS broth and incubated at 37 °C in a ZHTY-70 N orbital shaker (Shanghai Zhichu Instrument Co., Ltd., Shanghai, China) (Liu et al., 2019). Uninoculated MRS broth was used as a negative control. At 2-h intervals, aliquots of the bacterial suspension were collected, and the optical density at 600 nm (OD600) was measured using a TU-1900 UV–Vis spectrophotometer (Beijing Pursee Instrument Co., Ltd., China). The growth curve was plotted with incubation time as the abscissa and OD600 values as the ordinate to characterize the growth kinetics of *Lactobacillus* in MRS broth. The growth curve in RWL extract: The Activated *Lactobacillus reuteri* and *Lactobacillus plantarum* were mixed in a ratio of 1:1 (*v*/v), then 2.50 mL of mixed *Lactobacillus* liquid were transferred into 100 mL RWL extract prepared by 2.2.1. Incubated at 37 °C for 32 h in a shaker. The OD600 at 600 nm was detected at an interval of 2 h, and the growth curve in RWL extract was drawn.

#### 2.2.4 Freeze drying

According to the *Lactobacillus* culture method described in Section 2.2.3, RWL extract was added at a concentration of 30 % (*w*/w). After 26 h of cultivation, the *Lactobacillus* culture was combined with RWLEP obtained from Section 2.2.1. The proportions of RWLEP in the mixtures were 0 %, 5 %, 10 %, 20 %, 30 %, and 40 % (w/w). The mixtures were homogenized and then subjected to pre-freezing at −80 °C for 2 h, in accordance with the method described by Yang (Yang, 2015). Subsequently, the pre-frozen samples were freeze-dried using an FD 5–2.5E freeze dryer (Jinximeng Instrument Co., Ltd., Beijing, China) with a drying thickness of 1.0 cm and a drying time of 24 h. The resulting freeze-dried powder (FDP) was used to determine the survival rate of *Lactobacillus*.

#### 2.2.5 Spray drying

Take 25 g RWLEP prepared from 2.2.1 with 30 % RWL addition, put it in 100 mL ultrapure water, and homogenize it in an XHF-DY homogenizer (Ningbo Xinzhi Biotechnology Co., Ltd., Ningbo, China) at 10000–14000 rpm for 10 min, and then filter it with gauze to obtain spray drying protective matrix materials. According to the *Lactobacillus* culture method in 2.2.3, use RWL extract with 30 % RWL addition and mix the solution of *Lactobacillus* culture after 26 h with protective matrix materials. The proportion of protective matrix materials was 0 %, 2.5 %, 5.0 %, 7.5 %, 10.0 %, 12.5 % (*w*/w). After mixing evenly, the mixed solution was spray-dried using an LNB-9000Y spray dryer (Shanghai Haozhuang Instrument Co., Ltd., Shanghai, China). According to Gardiner (Gardiner et al., 2000) the air flow rate is 25 m^3^/h, the relative humidity is at 53 %. the temperature of the inlet air was 100 °C, the temperature of the outlet air was 50 °C–60 °C, the feeding speed was 30 rpm, and the fan frequency was 30–50 Hz. Spray-dried powder (SDP) was obtained to detect the survival rate of *Lactobacillus*.

### 2.3 Test methods

#### 2.3.1 Sugars composition and content analysis

The RWL extract was prepared using method 2.2.1 with 30 % RWL addition. The glutinous rice extract was prepared using method 2.2.1 with 30 % glutinous addition. The fermented RWL extract was the supernatant obtained by centrifuging the solution of RWL extract cultured with *Lactobacillus* using the method in 2.2.4. The method of sugar composition and content was based on previous research with slight modifications (Zhang, Pan, et al., 2020).

50 μL of each sample was mixed with 500 μL of methanol: isopropanol: water (3:3:2 *V*/V/V), vortex-mixed for 3 min, and sonicated for 30 min. The extract was centrifuged at 14,000 rpm under 4 °C for 3 min. 50 μL of the supernatant was mixed with 20 μL internal standard (1000 μg/mL) and evaporated under a nitrogen gas stream. The evaporated sample was transferred to the lyophilizer for freeze-drying. The residue was used for the further derivatization.

The derivatization method was as follows: the sample was mixed with 100 μL solution of methoxyamine hydrochloride in pyridine (15 mg/mL). The mixture was incubated at 37 °C for 2 h. Then 100 μL of BSTFA was added into the mixture and kept at 37 °C for 30 min after vortex-mixing. The mixture was analyzed by GC–MS after diluting to an appropriate concentration.

(1) GC–MS analysis

Agilent 7890B gas chromatograph coupled with a 7000D mass spectrometer with a DB-5MS column (30 m length × 0.25 mm i.d. × 0.25 μm film thickness, J&W Scientific, USA) was employed for GC–MS analysis of sugars. Helium was used as carrier gas at a flow rate of 1 mL/min. Injections were made in the split mode with a split ratio of 3:1, and the injection volume was 2 μL. The oven temperature was held at 150 °C for 1 min and then raised to 200 °C at 5 °C/min, raised to 300 °C at 16 °C/min, raised to 320 °C at 20 °C/min and held at the temperature for 5.5 min. All samples were analyzed in selective ion monitoring mode. The ion source and transfer line temperature were 230 °C and 280 °C, respectively.

#### 2.3.2 Relative survival rate of Lactobacillus

Accurately weighed 0.5 g of FDP and SDP in 4.5 mL of phosphate buffer (pH = 7.0) with a concentration of 0.2 M. The powder particles were stirred at 37 °C until all the powder particles were dissolved, and then the number of viable *Lactobacillus* was determined by plate counting method (Niu et al., 2019).

(1) $$\text{Relative survival rate}\;\left(\%\right)=\frac{N_{t}}{N_{o}}\times 100\%$$

Where N_0_ is the number of viable *Lactobacillus* (CFU / mL) in the solution of *Lactobacillus* culture before drying, and N_t_ is the number of viable *Lactobacillus* (CFU / mL) in the rehydrated solution.

#### 2.3.3 Electronic nose

The samples were accurately weighed 3.0 g in headspace vials and allowed to stand for 30 min at room temperature, sealed with caps. Three sets of parallels were made for each sample, and headspace sampling was used for the determination of flavor (André et al., 2019; Liu et al., 2016). The parameters of the electronic nose host were set as follows: data acquisition time of 120 s, data acquisition delay of 500 s, flow rate of 150 mL/min, and injection rate of 500 μL. The parameters of the autosampler were set as follows: incubation time of 360 s at 40 °Cand an injector temperature of 90 °C. Before and after measurements, the sensors were cleaned and standardized. The electronic nose system includes 27 sensors sensitive to distinct compounds, as follows: sn1 short chain alkanes; sn2 carbon containing substances; sn3 Hydrogen; sn4 Sulfur-containing compounds; sn5 nitrogen containing compounds; sn6 aldehydes and Ketones; sn7 super alkanes and combustible gases; sn8 liquefied petroleum gas; sn9 alkanes, alcohols, ketones, etc.; sn10 nitrogen-containing compounds and hydrogen; sn11 alkanes, carbon monoxide, etc.; sn12 specific organic solvents; sn13 short chain alkanes; sn14 short chain alkanes; sn15 nitrogen-sensitive compounds; sn16 sulfur-sensitive compounds; sn17 Hydrogen-containing substances; sn18 alcohol, some organic solvents; sn19 alcohols, aldehydes, ketones, benzenes; sn20 super alkanes; sn21 combustible gases; sn22 VOC; sn23 alcohols, aldehydes, ketones, benzenes; sn24 alkanes, alkenes, hydrogen; sn25 alkanes, CO, alkenes, aldehydes, NOx, ketones; sn26 organic solvents; sn27 sulfides, nitrides, and carbides.

#### 2.3.4 Analysis of GC-IMS

Three samples were tested using GC-IMS, i.e., fermentation broth (Sample 1) lyophilized bacterial powder (Sample 2) and spray-dried bacterial powder (Sample 3). The samples were kept sealed at room temperature. Prior to testing, the three samples (1 mL each) were placed into 20 mL headspace vials, which were sealed and placed into the injection tray for testing. The injection needle temperature was 85 °C and the incubation speed was 500 rpm. The column temperature was 60 °C and the carrier gas/drift gas was N2 at 45 °C. The analysis time was 30 min (GC conditions: 0–2 min: 150 mL/min of drift gas, 2 mL/min of carrier gas; 2–10 min: 150 mL/min of drift gas, 2 mL/min to 10 mL/min of carrier gas; 10–25 min: 150 mL/min of drift gas, 10 mL/min to 100 mL/min of carrier gas); (25–30 min: 150 mL/min of drift gas and 100 mL/min of carrier gas). The column model was MXT®-WAX (length: 30 m, inner diameter: 0.53 mm, film thickness: 1 μm). Qualitative analysis was performed using the built-in NIST and IMS databases within the software. Standard curves were established for each compound to conduct quantitative analysis. Fingerprint profiles were generated using the Gallery Plot plug-in, where each point represents a volatile organic compound. The color gradient reflects the compound's concentration: white indicates lower concentrations, red denotes higher concentrations, and deeper color intensity corresponds to greater concentration levels.

#### 2.3.5 Simulated gastrointestinal fluid tolerance test

0.1 g of FDP and SDP were weighed and added to 0.9 g of PBS solution, respectively, and dissolved for 10 min at 37 °C. The samples were added to 9.0 g of preheated simulated gastric fluid (or artificial intestinal juice) at 37 °C and incubated for 0 min, 30 min, 60 min, 90 min, 120 min and 150 min at 37 °C, 100 rpm in a water bath shaker (Bosnea et al., 2014). The gastric fluid (or artificial intestinal juice) treated samples were centrifuged at 4 °C, 10,000 rpm for 10 min, and the pH of the mixture should be adjusted to 6.0–8.0 before centrifugation to obtain the precipitate and redissolve it in 0.9 g of PBS solution to determine the survival *Lactobacillus* count by plate counting method.

#### 2.3.6 Storage stability

SDP-7.5 % and FDP-30 % (1.0 g each) were packaged in aluminum foil pouches (5 × 15 cm) and stored at −20 °C, 4 °C, or 20 °C. Viable *Lactobacillus* counts were determined on days 3, 7, 14, 21, 30, and 60 using the method described in Section 2.3.2 (Yang, 2015).

#### 2.3.7 Microstructure of FDP and SDP

The morphology of spray-dried rice wine lees powder, SDP-7.5 % RWLEP, and FDP-30 % RWLEP was analyzed using a MIRA scanning electron microscope (SEM) (TESCAN, China). Samples were sputter-coated with gold and imaged at 15 kV acceleration voltage the magnification is 2000× (Guo et al., 2018).

#### 2.3.8 Particle size distribution

According to Zhao Yuhan et al. (Zhao et al., 2022) a BT-9300HT laser particle size analyzer (Dandong Baite Instrument Co., Ltd., Dandong, China) was used to analyze the particle size of the spray dried rice wine lees powder, SDP-7.5 % RWLEP, FDP-30 % RWLEP. Take an appropriate amount of powder and dissolve it in ultrapure water. Transfer it to a beaker filled with distilled water for dispersion until the shading background intensity reaches 12 % to stop the dropping and test to obtain a particle size distribution curve. The refractive coefficient of distilled water is known to be 1.33.

#### 2.3.9 Data analysis

Data are expressed as mean ± standard deviation (*n* ≥ 3). Significant differences (*P* < 0.05) were evaluated by one-way ANOVA using SPSS 23.0.

## 3 Results and analysis

### 3.1 Sugars composition and content

**Fig. 1:**
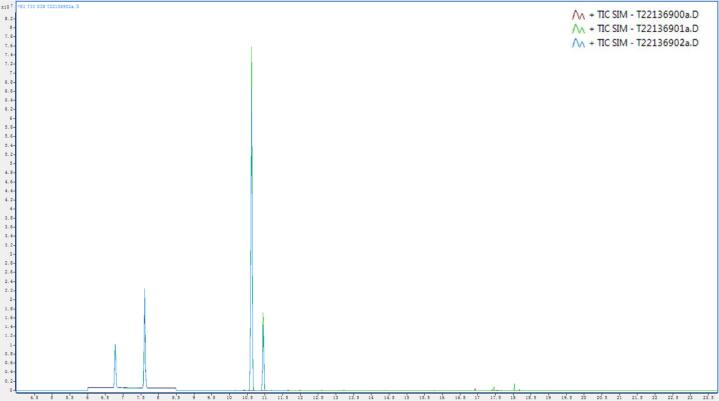
The TIC (Total Ion Current) chromatogram of sugar content in fermentation broth.

Determination of sugar composition and content in glutinous rice extract, RWL extract, and fermented RWL extract. The ion chromatograms are shown in Fig. 1. The results are presented in Table 1. A total of 24 sugars were detected, including 5 disaccharides, 18 monosaccharides and 1 trisaccharide. Glutinous rice extract was not fermented. RWL extract was mainly fermented by yeast during the preparation of rice wine. The fermented RWL extract was fermented by *Lactobacillus* on the basis of RWL extract, so the sugar composition and content of the three samples were quite different. Compared with glutinous rice extract, the newly added sugars in RWL extract and fermented RWL extract include Deoxyglucose, d-Ribose, D-Arabinose and D-Ribono-1,4-lactone. Compared with glutinous rice extract, the sugar disappeared in RWL extract and fermented RWL extract were D-Glucuronic acid, Methyl β-D-galactopyranoside and 2-Acetamido-2-deoxy-d-glucopyranose.

**Table 1:** The sugars composition and content of the RWL extract, glutinous rice extract and fermented RWL extract.

Compounds	Class	Molecular Weight	Glutinous rice extract	RWL extract	Fermented RWL extract	CAS
			/×10^−3^ mg/mL			
Phenylglucoside	Disaccharide	256.1	0.34	0.31	0.21	1464-44-4
Cellobiose		342.1	/	8.11	/	528-50-7
Trehalose		342.1	1.19	253.17	53.38	99-20-7
Maltose		342.1	7.52	443.45	13.08	69-79-4
Sucrose		342.1	143.73	0.39	/	57-50-1

Deoxyglucose	monosaccharide	164.1	/	1.61	1.12	154-17-6
D-Glucuronic acid		194.0	5.26	/	/	6556-12-3
Methyl beta-D-galactopyranoside		194.1	0.57	/	/	1824-94-8
D-Arabinitol		152.1	2.17	2.56	2.62	488-82-4
2-Acetamido-2-deoxy-d-glucopyranose		221.1	2.35	/	/	7512-17-6
d-Ribose		150.1	/	12.08	16.68	50-69-1
Inositol		180.1	11.04	/	1.40	87-89-8
D-Arabinose		150.1	/	18.67	5.06	10323-20-3
D-Xylulose		150.1	0.27	1.42	9.43	551-84-8
D-Xylose		150.1	0.58	1.51	1.54	58-86-6
D-Sorbitol		182.1	1.72	11.32	10.15	50-70-4
D-Mannose		180.1	3.40	86.44	78.12	3458-28-4
Levoglucosan		162.1	6.22	7.11	6.90	498-07-7
Glucose		180.1	41.33	33,936.32	29,972.54	50-99-7
D-Galacturonic acid		194.0	1.58	5.11	4.53	685-73-4
D-Galactose		180.1	1.79	3.03	2.32	59-23-4
d-Fructose		180.1	6.76	56.24	2.15	7660-25-5
D-Ribono-1,4-lactone		148.0	/	134.52	131.82	5336-08-3

Raffinose	trisaccharide	504.2	8.15	3.88	3.94	512-69-6

Compared with fermented RWL extract, most of the carbon source in RWL extract was absorbed after fermentation of RWL extract by *Lactobacillus*, indicating that the *Lactobacillus* could utilize cellobiose, maltose, sucrose, D-arabinose, glucose, D-galactose, and d-fructose. This is in general agreement with the studies by Wang Jia (Wang et al., 2023). It was proven that *Lactobacillus plantarum* and *Lactobacillus reuteri* have a wide range of carbon sources that can be utilized in RWL extract, especially for glucose, maltose, and trehalose.

### 3.2 The growth curves of *Lactobacillus plantarum* and *Lactobacillus reuteri*

**Fig. 2:**
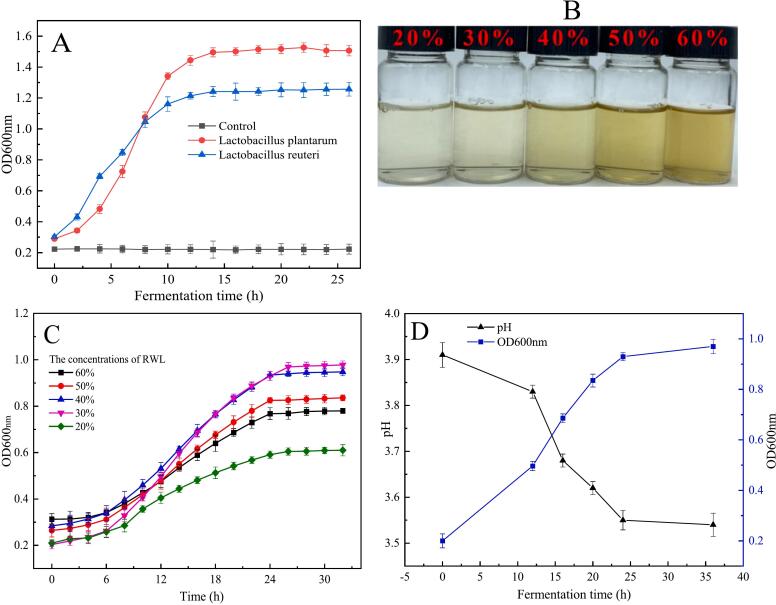
A represents the growth curve of *Lactobacillus plantarum* and *Lactobacillus reuteri* in MRS broth； B represents the appearance of Rice wine lees (RWL) extract with different concentrations；C represents the growth curve of *Lactobacillus* in different concentrations of RWL extract (The ratio of two kinds of *Lactobacillus* was 1:1(*v*/v))； D represents OD600 value and pH value in 30 % RWL extract.

The growth curve of *Lactobacillus plantarum* and *Lactobacillus reuteri* in MRS broth is shown in Fig. 2A. It can be seen that *Lactobacillus plantarum* grows slowly in 0–4 h, which belongs to the delayed period, and the OD600 value increased rapidly in 4–10 h, when the proliferation rate was accelerated and enters the logarithmic period of growth (Li et al., 2021) and reaches the stable period after 12 h, when the OD600 value reaches 1.56, and the number of *Lactobacillus* does not vary much from now on. *Lactobacillus reuteri* was in the logarithmic period of growth in 2–10 h (Liu, 2018) with a faster growth rate. After 12 h, entered the growth stabilization period, and the OD600 value was basically around 1.2. *Lactobacillus plantarum* and *Lactobacillus reuteri* had different growth rates, and the final OD600 values were different, but all of them entered into the growth stabilization period at about 12 h.

As shown in Fig. 2B, the color of the RWL extract gradually changed from light yellow to dark yellow with the increase of RWL addition. This may be because the more RWL was added, the more sugar content in the extract, and the Maillard reaction occurs in the extract after high temperature and high-pressure sterilization (Ou, 2005). The more sugar content in the extract, the higher the reaction degree and the darker the color. The growth curve of *Lactobacillus* in different concentrations of RWL extract is shown in Fig. 2C. The OD600 value increased with the fermentation time and reached the stabilization period of fermentation at 26 h. Then, in the subsequent freeze drying and spray drying, 26 h was selected as the end point of fermentation and its OD600 value was measured. The OD600 values of *Lactobacillus* fermented in different concentrations of RWL extract were different, and with the increase of RWL concentration addition, the OD600 values showed a tendency to increase and then decrease, and the OD600 values reached the highest when the RWL addition reached 30 %.

The OD600 value and pH value in 30 % RWL extract are shown in Fig. 2D. It can be seen that the OD values and pH values were inversely proportional to each other with the increase in fermentation time, which was because the higher the density of the *Lactobacillus*, the higher the fermentation, and the higher the acid production of the *Lactobacillus*, which led to the decrease of the overall pH within the solution.

### 3.3 Effect of drying methods on the relative survival rate

**Fig. 3:**
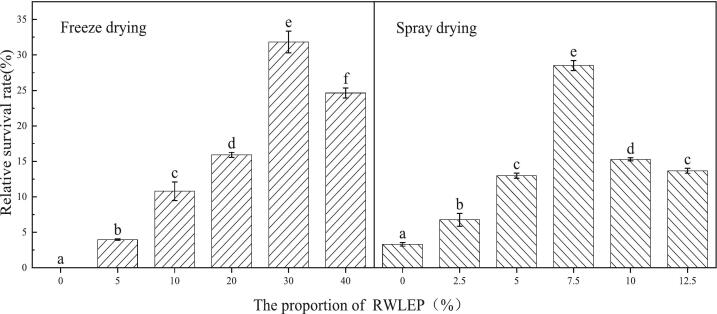
Effect of different drying methods on the relative survival rate of *Lactobacillus.*

The relative survival rate of *Lactobacillus* using freeze drying and spray drying are shown in Fig. 3. It can be seen that the relative survival rate increased significantly with the increase of RWLEP dosage in the freeze drying, this is mainly because RWL is rich in protein. During vacuum freeze-drying, the protein in the lees forms a protective protein membrane around bacterial cells, preventing cell damage. Other components in the lees, such as carbohydrates, also protect the bacteria, which showed that RWLEP played a protective role. When the addition amount was 30 %, the relative survival rate reached 31.82 %, and the relative survival rate decreased with the addition of RWLEP (>30 %), which may be due to the increase of solid content in RWLEP and the high viscosity of the RWLEP, which was not conducive to the volatilization of water in the freeze drying process, caused more damage to *Lactobacillus* in the freeze drying process, and reduced the survival rate.

In SDP, some lactic acid bacteria can still survive without RWLEP, which may be due to the incomplete utilization of polysaccharides in fermentation RWL extract, which formed a certain protective effect during spray drying. The relative survival rate of the *Lactobacillus* exhibited a certain increment with the addition of RWLEP protective matrix material. When the proportion of RWLEP added was 7.5 %, the survival rate was about 28.5 %. The employment of a protective matrix in the spray drying process can reduce the incompleteness of cell walls and protein structures caused by high temperatures, and can effectively prevent mechanical damage to the cell membranes (Dong-Hwan et al., 2017). When the proportion of RWLEP added reached 10 %, the relative survival rate of the *Lactobacillus* decreased, which may be attributed to excessive RWLEP. Excessive RWLEP can increase the sugar content in the *Lactobacillus* suspension, thereby being unfavorable for *Lactobacillus* atomization (Liu et al., 2020) or increase the viscosity of the feed solution, prolong the thermal contact time of *Lactobacillus* cells during the drying process. Therefore, the RWLEP concentration of 7.5 % was selected as the protective matrix for spray drying in this experiment.

### 3.4 Electronic nose

**Fig. 4:**
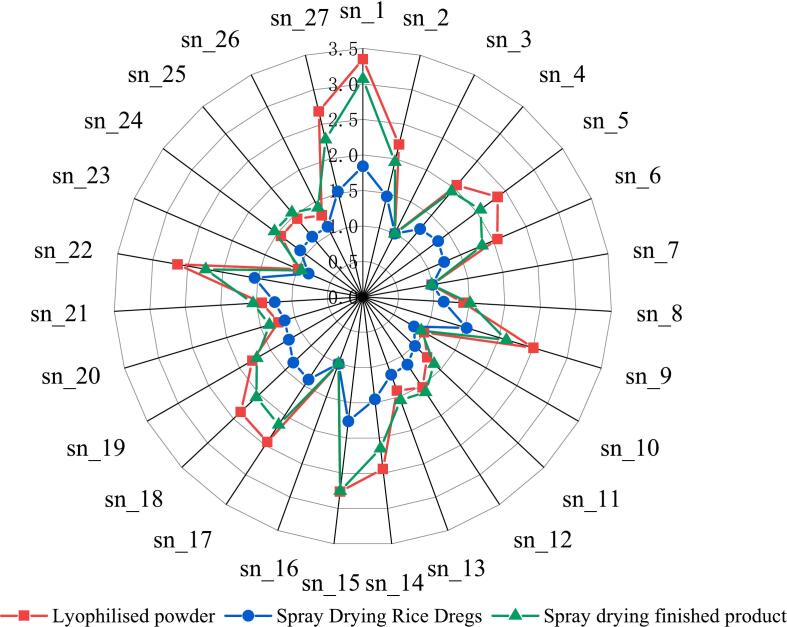
Characteristic radar map of electronic nose detection of powder.

As shown in Fig. 4, the response values of rice residue treated by different drying methods are different, spray drying has significantly lower response values than vacuum freeze drying, and spray dried rice residue has the lowest response values for all sensors.

The most changed sensors were sn1, sn2, sn4, sn5, sn6, sn9, sn14, sn15, sn16, sn18, sn19, sn23, sn27. These sensors corresponded to short-chain alkanes (propane); carbon-containing substances (alcohols, smoke, isobutane, formaldehyde, etc.); sulfur-containing compounds (hydrogen sulphide, sulphide, etc.); and nitrogen-containing compounds (ammonia, amines, etc.); aldehydes and ketones (toluene, acetone, ethanol, etc.); alkanes, alcohols, ketones, etc. (toluene, formaldehyde, benzene, alcohol, acetone, etc.); short-chain alkanes (Methane, etc.); nitrogen-sensitive compounds (ammonia, amines, etc.); sulfur-sensitive compounds (hydrogen sulphide, sulphide, etc.); alcohol, some organic solvents (aromatic hydrocarbons, aliphatic hydrocarbons, aliphatic hydrocarbons, etc.); alcohols, aldehydes, ketones, benzenes (alcohols, ketones, aromatic compounds, etc.);alkanes, olefins, aromatic hydrocarbons (butanes, butenes, etc.); sulfides, nitrides, and carbides (those causing smoke or cooking odors etc.) which indicates that high temperature during spray drying has a significant effect on these volatile substances.

### 3.5 Analysis of GC-IMS

#### 3.5.1 Direct comparison of sample VOC differences

**Fig. 5:**
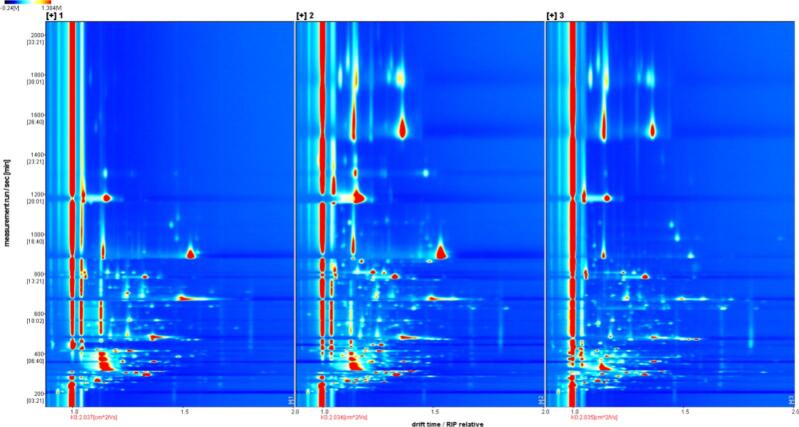
GC-IMS spectrum (top view).

The signals are not significant in the samples. In order to further compare the differences between different samples, the bacterial liquid and two samples of bacterial powder were analyzed, and the results are shown in Fig. 5. Each characteristic peak in the spectrum represents a volatile organic component, The differential comparison mode was employed, and according to the principle of difference spectra, the spectrum of the sample with fewer volatile components was selected as a reference, and the spectrum of the other samples was deducted from the reference to carry out the difference spectra analysis. There were significantly more volatile flavor compounds in the vacuum freeze-dried samples than in the spray-dried ones, indicating that vacuum freeze-drying has the advantage of retaining volatile flavor compounds in the samples.

According to Fig. 5, the gas phase ion mobility spectra of the samples show the variation of VOC species and concentrations in the three samples. As can be seen from the above figure, most of the signals occur at drift times 1.0–1.5 and retention times 0–800 s.

#### 3.5.2 Qualitative analysis of samples for VOCs

The three samples in this study were fermented bacterial liquid, freeze-dried bacterial powder, and spray-dried bacterial powder. The results of the identification of VOCs in the bacterial powders with different drying methods are shown in Table 2, and a total of 36 VOCs were identified in all the samples, including 6 aldehydes, 3 ketones, and 11 alcohols.

**Table 2:** List of qualitative compounds in gas phase ion mobility spectrum.

No.	Chemical compound	CAS number	Molecular formula	Molecular mass	Relative retention index	Retention time	Relative migration time
1	2-Methylpropanol	C78831	C_4_H_10_O	74.1	1101.9	482.966	1.17513
2	2-Methylpropanol	C78831	C_4_H_10_O	74.1	1102.1	483.365	1.36931
3	3-Methyl-1-butanol	C123513	C_5_H_12_O	88.1	1216.8	678.774	1.24118
4	3-Methyl-1-butanol	C123513	C_5_H_12_O	88.1	1217.3	679.41	1.49087
5	3-Hydroxy-2-butanone	C513860	C_4_H_8_O_2_	88.1	1291.4	786.225	1.06427
6	3-Hydroxy-2-butanone	C513860	C_4_H_8_O_2_	88.1	1291.8	786.861	1.33152
7	Ethyl lactate	C97643	C_5_H_10_O_3_	118.1	1354	890.768	1.13823
8	Ethyl lactate	C97643	C_5_H_10_O_3_	118.1	1355.8	893.99	1.53735
9	acetic acid	C64197	C_2_H_4_O_2_	60.1	1495.3	1180.713	1.05078
10	acetic acid	C64197	C_2_H_4_O_2_	60.1	1497.3	1185.546	1.15731
11	benzaldehyde	C100527	C_7_H_6_O	106.1	1544.7	1303.135	1.15095
12	benzaldehyde	C100527	C_7_H_6_O	106.1	1546.6	1307.967	1.47057
13	furfural	C98011	C_5_H_4_O_2_	96.1	1472.2	1127.557	1.09212
14	ethanol	C64175	C_2_H_6_O	46.1	961.1	338.175	1.13447
15	acetone	C67641	C_3_H_6_O	58.1	853.7	273.915	1.10886
16	butanol	C71363	C_4_H_10_O	74.1	1152.7	569.205	1.18435
17	butanol	C71363	C_4_H_10_O	74.1	1153.5	570.735	1.37979
18	isoamyl acetate	C123922	C_7_H_14_O_2_	130.2	1134.3	536.31	1.74776
19	ethyl acetate	C141786	C_4_H_8_O_2_	88.1	898.6	299.16	1.33666
20	2-Butanone	C78933	C_4_H_8_O	72.1	915.3	309.105	1.245
21	Methyl acetate	C79209	C_3_H_6_O_2_	74.1	836.3	264.713	1.19583
22	terbutyl alcohol	C75650	C_4_H_10_O	74.1	926.1	315.682	1.32166
23	2-Methylbutyraldehyde	C96173	C_5_H_10_O	86.1	926.9	316.197	1.39813
24	2-Propanol	C67630	C_3_H_8_O	60.1	948.4	329.84	1.23454
25	Ethyl propionate	C105373	C_5_H_10_O_2_	102.1	969.9	343.998	1.44943
26	1-Propanol	C71238	C_3_H_8_O	60.1	1050	418.635	1.11096
27	1-Propanol	C71238	C_3_H_8_O	60.1	1049.6	418.177	1.25603
28	butyl acetate	C123864	C_6_H_12_O_2_	116.2	1074.6	447.504	1.23668
29	Isoamyl formate	C110452	C_6_H_12_O_2_	116.2	1074.6	447.504	1.26892
30	3-Methyl-3-buten-1-ol	C763326	C_5_H_10_O	86.1	1254.1	730.535	1.17036
31	2-Methylpropanal	C78842	C_4_H_8_O	72.1	828.8	260.864	1.28295
32	Heptaldehyde	C111717	C_7_H_14_O	114.2	1192	646.17	1.33583
33	Hexyl alcohol	C111273	C_6_H_14_O	102.2	1367.3	914.637	1.32741
34	Hexyl butyrate	C2639636	C_10_H_20_O_2_	172.3	1403.8	983.702	1.4791
35	2-Ethyl-3-methylpyrazine	C15707230	C_7_H_10_N_2_	122.2	1413.3	1002.509	1.17995
36	Propionic acid	C79094	C_3_H_6_O_2_	74.1	1567.3	1363.125	1.10923
37	1,3-Butanediol	C107880	C_4_H_10_O_2_	90.1	1625	1529.273	1.14028
38	1,3-Butanediol	C107880	C_4_H_10_O_2_	90.1	1620.3	1515.015	1.3691
39	4-Hydroxybutyrate	C96480	C_4_H_6_O_2_	86.1	1704.4	1791.751	1.08615
40	3-Methyl-2-butanol	C598754	C_5_H_12_O	88.1	1080.1	454.199	1.41558
41	2-Heptanone	C110430	C_7_H_14_O	114.2	1188.3	638.499	1.62762
42	1-Penten-3-ol	C616251	C_5_H_10_O	86.1	1168.7	599.423	0.94012
43	2-Methylpyrazine	C109080	C_5_H_6_N_2_	94.1	1271.4	755.809	1.07659
44	2-Methylpyrazine	C109080	C_5_H_6_N_2_	94.1	1271.4	755.809	1.09047
45	Hydroxyacetone	C116096	C_3_H_6_O_2_	74.1	1305.9	809.264	1.22989
46	Hexanal	C66251	C_6_H_12_O	100.2	1096.7	475.207	1.56299

**Fig. 6:**
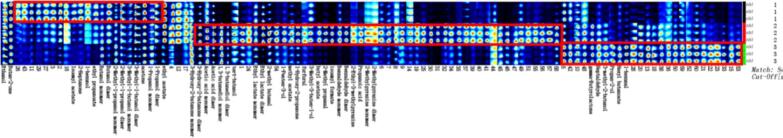
Gallery Plot.

The fingerprints of volatile organic compounds in the samples are shown in Fig. 6, from which it is found that the 3 samples can be differentiated by the type and concentration of volatile substances, which can be observed to be different from each other through the above figure.

As shown in Fig. 6, the main substances in Sample No. 1 were: isoamyl acetate, 2-heptanone, hexanol, ethyl propionate, butanol, and acetone, and in higher concentrations compared to the other two samples. Isoamyl acetate is a liquid with a banana odour and is volatile; 2-heptanone has a characteristic banana-like aroma and a slight medicinal aroma; ethyl propionate is an organic substance, a colorless liquid with a pineapple aroma; butanol has a specific odour; and acetone has a specific pungent odour.

As shown in Fig. 6, the main substances in sample No. 2 are: ethyl lactate, 3-methyl-3-buten-1-ol, 2-methylpropanal, methyl acetate, tert-butanol, butyl acetate, isoamyl formate, benzaldehyde, furfural, 2-methylpyrazine, 3-methylpyrazine, propionic acid, 1-penten-3-ol, 1-hydroxy-2-propanone, 2-methylpyrazine, and 1,3-butanediol, etc., and the concentration of them is higher compared to the other two samples. Higher concentrations compared to the other two samples. Ethyl lactate has the aroma of rum, fruit and cream; 3-methyl-3-buten-1-ol has an unpleasant odour; 2-methylpropanal has a special strong irritating odour; methyl acetate has an aromatic odour; tert-butanol has a camphor-like odour; butyl acetate has a fruity aroma; isoamyl formate has a plum fruity odour; benzaldehyde has a bitter almond odour; furfural has an almond odour; and 2-and 3-methylpyrazine have a roasted protein odour. Methylpyrazine has a toasted protein odour and a cocoa-nut odour; propionic acid has an unpleasant rancid pungent odour; 1-penten-3-ol has a fruity, vegetable, and horseradish odour; 1-hydroxy-2-propanone has a weak odour; 2-methyl pyrazine has a nutty, potato flake, and chocolatey odour; and 1,3-butanediol has virtually no odour.

As shown in Fig. 6, the main substances in sample No. 3 were: heptanal, 3-methyl-2-butanol, propylene glycol, hexyl butyrate and hexanol and were in higher concentrations compared to the other two samples. Heptaldehyde has a pungent, fruity, wax-like odour; 3-methyl-2-butanol has a camphor-like odour; propylene glycol is virtually tasteless and odourless; hexyl butyrate has a fruity, apricot-like odour; and hexanol has a camphor-like odour.

By comparing the flavor substances of the samples, Sample 2 and Sample 3, the two drying methods, vacuum freeze-drying and spray-drying, had a significant effect on the volatile substances, with vacuum freeze-drying preserving the odour species to a greater extent, which is consistent with the findings of Song (Song et al., 2017) The number of volatile substances in the samples was significantly reduced after spray drying, this phenomenon may be due to the high temperature of the air outlet of spray drying, which resulted in the loss of flavor.

### 3.6 Simulated gastric fluid tolerance test

After being absorbed by the human body, probiotics need to pass through the stomach, small intestine, and other parts to reach the large intestine to play a beneficial role. Gastric fluid and intestinal fluid have an adverse impact on the survival of probiotic strains. The main components of human gastric fluid include gastric acid and pepsin, which are secreted substances with antimicrobial properties. As a probiotic must be able to tolerate gastric fluid. The FDP-30 % RWLEP and SDP-7.5 % RWLEP were tested to simulate gastric fluid.

**Fig. 7:**
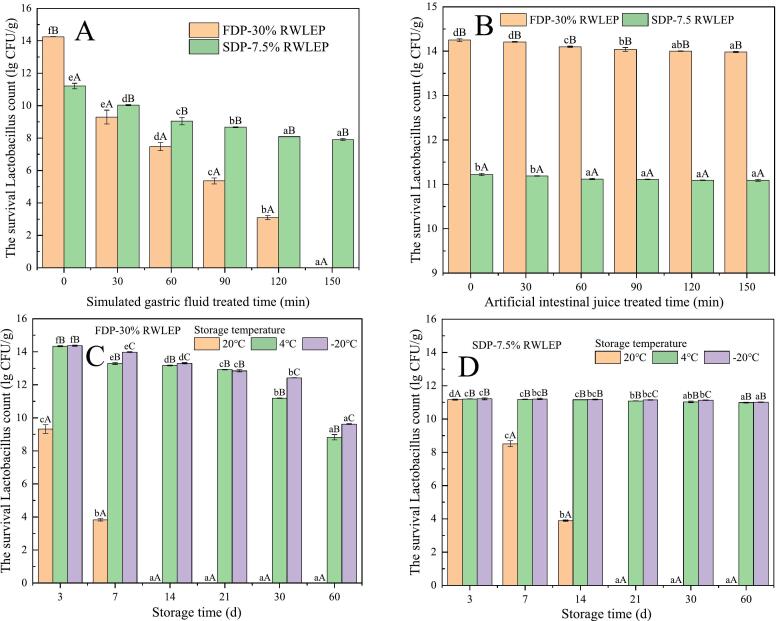
A represents the number of lactobacilli surviving in freeze-dried powder (FDP)-30 % RWLEP and Spray-dried powder (SDP)-7.5 % Rice wine lees extra precipitate (RWLEP) cultured in simulated gastric fluid from 0 min to 150 min (a–f). b represents the number of lactobacilli surviving in FDP-30 % RWLEP and SDP-7.5 % RWLEP cultured in artificial intestinal fluid from 0 min to 150 min (a–d). c represents the storage stability of FDP-30 % RWLEP (a-f). d represents the storage stability of SDP-7.5 % RWLEP (a–d). denotes the storage stability of FDP-30 % RWLEP (a–f). d denotes the storage stability of SDP-7.5 % RWLEP (a–d). Where letters between different samples indicate significant differences (*P* < 0.05).

The survival count of *Lactobacillus* the FDP-30 % RWLEP and SDP-7.5 % RWLEP incubated in simulated gastric fluid from 0 min to 150 min are presented in Fig. 7A. The initial *Lactobacillus* count of the FDP-30 % RWLEP were higher than those of the SDP-7.5 % RWLEP. The FDP-30 % RWLEP and SDP-7.5 % RWLEP showed a downward trend in the survival *Lactobacillus* count with the increase of simulated gastric fluid treated time. The survival *Lactobacillus* count of the FDP-30 % RWLEP decreased from 14.25 lg CFU/g to 0.00 lg CFU/g during the simulation of gastric fluid, which may be attributed to the fact that lyophilized powder did not provide a good encapsulation of *Lactobacillus*, failing the strain to survive in simulated gastric fluid. The survival *Lactobacillus* count of SDP-7.5 % RWLEP decreased from 11.21 lg CFU/g to 7.90 lg CFU/g. The survival *Lactobacillus* count was 7.90 lg CFU/g after simulated gastric fluid treatment for 150 min. This shows that during spray drying, the proteins and polysaccharides in rice wine lees form a physical barrier. This barrier effectively blocks the penetration of simulated gastric fluid, protecting the bacterial cells within the structure, which proved that the RWLEP played a certain protective effect on the *Lactobacillus* when spray drying.

### 3.7 Artificial intestinal juice tolerance test

The Intestinal juice contains digestive enzymes that are not favorable to the growth of the probiotics, as well as a high osmotic pressure environment. The FDP-30 % RWLEP and SDP-7.5 % RWLEP were added to the artificial intestinal juice, and the survival *Lactobacillus* count after treatment for 150 min are shown in Fig. 7B. It can be seen that the intestinal juice did not have significant effect on the survival *Lactobacillus* count of the FDP-30 % RWLEP and SDP-7.5 % RWLEP, and the FDP-30 % RWLEP decreased only by 0.5 lg CFU/g, and the survival *Lactobacillus* count were still high. The SDP-7.5 % RWLEP only decreased by 0.2 lg CFU/g, indicating that the FDP-30 % RWLEP and SDP-7.5 % RWLEP were better tolerated in intestinal juice, The addition of RWL as a protectant provides a certain degree of protection for both samples.

### 3.8 Storage stability

The temperature, humidity, water content and packaging materials will have a certain impact on the preservation period during the preservation of probiotics powder. In the experiment, the FDP-30 % RWLEP and SDP-7.5 % RWLEP were packaged with aluminum foil, and then the survival *Lactobacillus* count was detected at −20 °C, 4 °C and 20 °C respectively.

As shown in Fig. 7C, the survival *Lactobacillus* count of SDP-7.5 % RWLEP decreased rapidly at 20 °C and decreased to 0 % after 21 days. This phenomenon is likely due to the high dispersion of the freeze-dried powder under ambient conditions, which may disrupt cellular homeostasis or exhaust energy reserves, ultimately leading to bacterial death. These results indicate that ambient temperature is unsuitable for probiotic storage, as temperature exerts a significant influence on the viability of lactic acid bacteria strains.

However, there was no significant difference in the survival *Lactobacillus* count stored at −20 °C and 4 °C, and the trend was stable in the late stage, with no obvious downward trend. This stability can be attributed to the low-temperature environment substantially reducing the metabolic rate of the probiotics, inducing a dormant state characterized by minimal metabolic activity. In this state, biochemical reactions within cells slow down, energy consumption is drastically reduced, and cells only maintain essential physiological functions. Consequently, the probiotics retain relatively stable viability over extended periods, with minimal fluctuations in survival rates.

It can be seen from Fig. 7D that compared with SDP-7.5 % RWLEP, FDP-30 % RWLEP was not resistant to storage. After 2 months of storage, the survival *Lactobacillus* count on the whole shows a downward trend with the extension of storage time, which may be due to the lack of tight binding between *Lactobacillus* and protective agents (RWLEP) in the FDP-30 % RWLEP, *Lactobacillus* are more susceptible to environmental factors. High temperature causes some *Lactobacillus* to lose metabolic balance and die. The survival *Lactobacillus* count of FDP-30 % RWLEP at 4 °C and − 20 °C decreased in varying degrees, which was slower than that at 20 °C. The effect of FDP-30 % RWLEP stored at 4 °C and − 20 °C was better. The survival *Lactobacillus* count of FDP-30 % RWLEP stored at 4 °C for 2 months was 8.83 lg CFU/g, and that at −20 °C was 9.62 lg CFU/g, which was higher than the international Dairy Federation (IDF) recommends a minimum of 10^7^ live probiotic bacterial cells per gram or milliliter of product at the time of consumption (Corona-Hernandez et al., 2013; Song et al., 2017).

The survival *Lactobacillus* count of SDP-7.5 % RWLEP at 4 °C after 60 days of storage remained at 10.99 lg CFU/g, and the survival *Lactobacillus* count at −20 °C remained at 11.01 lg CFU/g. It can be seen that the lower the storage temperature, the better the storage effect, which was consistent with the storage rules of other *Lactobacillus* spray-dried powder and freeze-dried powder reported in the literature (Sun et al., 2020). Under low-temperature conditions, spray-dried powder can maintain a high survival *Lactobacillus* count at −20 °C or 4 °C for a long time. In conclusion, probiotics are more suitable for low-temperature preservation, and normal-temperature preservation is not conducive to maintaining the *Lactobacillus* count. Considering the storage cost, it is more appropriate to choose 4 °C for preservation.

### 3.9 The microstructure of the FDP and SDP

**Fig. 8:**
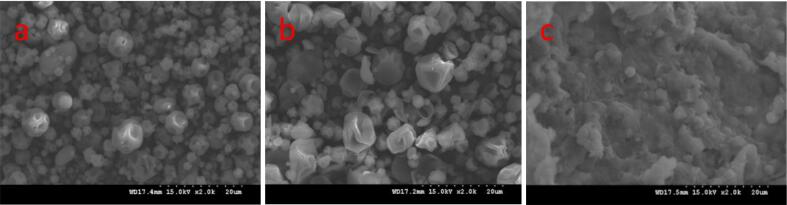
SEM of probiotic powder: (a) Spray dried rice wine lees powder; (b) SDP-7.5 % RWLEP; (c) FDP-30 % RWLEP.

The scanning electron microscope images of the FDP and SDP are shown in Fig. 8. A surface morphology study of the SDP (spray-dried rice wine lees powder and SDP-7.5 % RWLEP) showed a spherical collapsed shape and a smooth surface and the diameters of SDP varied from several micrometers to about 50 μm. Surface collapse can be caused by rapid evaporation of the water (Giuseppina et al., 2023). The surface of the FDP-30 % RWLEP was concave and finely crystalline, a phenomenon that may be due to solid-to-gaseous sublimation (Zhang, Yuan, et al., 2020), a structure that provides some protection to the encapsulated material (Charikleia & Constantina, 2014).

### 3.10 Particle size distribution

**Fig. 9:**
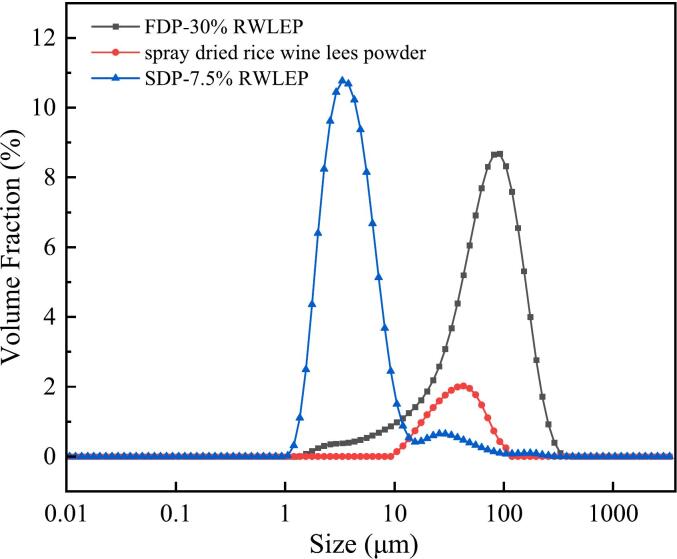
Particle size distribution.

The particle size distribution of FDP-30 % RWLEP, spray-dried rice wine lees powder, and SDP-7.5 % RWLEP are shown in Fig. 9. After fermentation, compared to FDP-30 % RWLEP, SDP-7.5 % RWLEP exhibited significantly smaller and more uniform particle sizes, the powder particles are further reduced in size, forming smaller spherical granules with a more loosely packed structure, consistent with SEM observations. These findings suggest that SDP-7.5 % RWLEP may possess enhanced solubility and dispersibility, making it suitable for applications in meal replacement powders, beverages, and other functional food products.

## 4 Conclusions

This study characterized the sugar composition and content of RWL through GC–MS analysis. *Lactobacillus plantarum* and *Lactobacillus reuteri* were inoculated in the liquid medium of RWL, and the test showed that the liquid medium of RWL promoted the growth of *Lactobacillus*. Finally, the cultured samples were dried and the results showed that RWLEP protective agent had a protective effect on *Lactobacillus* in FDP and SDP. The dried probiotic powder had better simulated gastric fluid tolerance, possesses favorable storage characteristics. It is hypothesized that probiotic powders could be used in the processing of grain meal replacement powders, beverages and other food products. The results and discussion of this study provide some references for the full utilization of rice wine lees and exploitation of its commercial value.

## CRediT authorship contribution statement

**Liya Tian:** Writing – original draft, Formal analysis, Data curation. **Xing Wang:** Formal analysis, Data curation. **Qingyun Lyu:** Methodology, Formal analysis, Data curation. **Lijie Zhu:** Supervision, Conceptualization. **Lei Chen:** Supervision. **Xi Chen:** Supervision, Conceptualization. **Wenping Ding:** Supervision, Resources, Project administration.

## Declaration of competing interest

The authors declare that they have no known competing financial interests or personal relationships that could have appeared to influence the work reported in this paper.

## Data Availability

Data will be made available on request.

## References

[bb0005] André B., Lotti E., Marie A., Paula A., Ricardo A., Simon B., Paul S.R. (2019). INFOGEST static in vitro simulation of gastrointestinal food digestion. Nature Protocols.

[bb0010] Bosnea L.A., Moschakis T., Biliaderis C.G. (2014). Complex coacervation as a novel microencapsulation technique to improve viability of probiotics under different stresses. Food and Bioprocess Technology.

[bb0015] Charikleia C., Constantina T. (2014). Thermooxidative stability of fennel oleoresin microencapsulated in blended biopolymer agents. Journal of Food Science.

[bb0020] Corona-Hernandez R.I., Álvarez-Parrilla E., Lizardi-Mendoza J., Islas-Rubio A.R., Rosa L.A., Wall-Medrano A. (2013). Structural stability and viability of microencapsulated probiotic bacteria: A review. Comprehensive Reviews in Food Science and Food Safety.

[bb0025] Dong-Hwan K., Sae-Byuk L., Heui-Dong P. (2017). Effect of air-blast drying and the presence of protectants on the viability of yeast entrapped in calcium alginate beads with an aim to improve the survival rate. Applied Microbiology and Biotechnology.

[bb0030] Du T., Lei A., Zhang N., Zhu. (2022). The beneficial role of probiotic Lactobacillus in respiratory diseases. Frontiers in Immunology.

[bb0035] Giuseppina G., Roberta R., Cristina M., Laura B., Elena P., Alfredo B., Stefano M. (2023). Microencapsulation by a spray drying approach to produce innovative probiotics-based products extending the shelf-life in non-refrigerated conditions. Molecules.

[bb0040] Guo Q., Ma Q., Xue Z. (2018). Studies on the binding characteristics of three polysaccharides with different molecular weight and flavonoids from corn silk (Maydis stigma). Carbohydrate Polymers.

[bb0045] He Z., Liu G., Qiao Z., Cao Y., Song M. (2021). Novel angiotensin-I converting enzyme inhibitory peptides isolated from rice wine lees: Purification, characterization, and structure-activity relationship. Frontiers in Nutrition.

[bb0050] Hong C., Ning S., Jiani R., Shuang Y., Yue C., Zhen P. (2024). Biotransformation characteristics of urate-lowering probiotic fermented apple juice and potential regulatory mechanisms for ameliorating hyperuricemia via mediating gut microbiota and metabolic pathways. Food Chemistry.

[bb0055] Hu Y., Pan L., Dun Y., Peng N., Liang Y., Zhao S. (2014). Conversion of yellow wine lees into high-protein yeast culture by solid-state fermentation. Biotechnology, Biotechnological Equipment.

[bb0060] Huang R., Tao X., Wan C. (2015). In vitro probiotic characteristics of *Lactobacillus plantarum* ZDY 2013 and its modulatory effect on gut microbiota of mice. Journal of Dairy Science.

[bb0065] Kalam A.M.A., Manobendro S., Tiejun L., Jie Y. (2018). Probiotic species in the modulation of gut microbiota: An overview. BioMed Research International.

[bb0070] Ke L., Wang L., Li H., Lin H., Zhao L. (2014). Molecular identification of lactic acid bacteria in Chinese rice wine using species-specific multiplex PCR. European Food Research and Technology.

[bb0075] Li H., Jiao A., Wei B., Wang Y., Wu C., Jin Z., Tian Y. (2013). Porous starch extracted from Chinese rice wine vinasse: Characterization and adsorption properties. International Journal of Biological Macromolecules.

[bb0080] Liu C., Zuo C., Peng J., Chen J., Tu K., Pan L. (2022). Response surface optimization of the fermentation process of tomato juice by *Lactobacillus plantarum* and its quality evaluation. Science and Technology of Food Industry.

[bb0085] Liu D. (2018).

[bb0090] Liu D., Hu M., Liu J., Zhu J., Li Y. (2016). Research of growth factors in high cell-density culture of Lactobacillus reuteri LT018. Science and Technology of Food Industry.

[bb0095] Liu R., Li Z., Mao S., Liang S., Zhao Y., Wang Y. (2020). Preparation and characterization of *Lactobacillus plantarum* microcapsules by compounding and multi-layer encapsulation. Science and Technology of Food Industry.

[bb0100] Liu S., Hou M., Chen X., Chao S., Yang Q. (2019). Effect of resistant starch on the proliferation of probiotics. Food Science and Technology.

[bb0105] Mohamadzadeh M., Fazeli A., Shojaosadati S.A. (2024). Polysaccharides and proteins-based bionanocomposites for microencapsulation of probiotics to improve stability and viability in the gastrointestinal tract: A review. International Journal of Biological Macromolecules.

[bb0110] Mu Q., Tavella V.J., Luo X.M. (2018). Role of *Lactobacillus reuteri* in human health and diseases. Frontiers in Microbiology.

[bb0115] Niu C., Miao X., Niu H., Liu J., Sun R., Li D. (2019). Development of composite vacuum freeze-dried probiotic fermentation protectants. Light industry. Science and Technology.

[bb0120] Ou Y. (2005). Cognition for caramel. Chinese Condiment.

[bb0125] Rani S., Pooja K., Pal G.K. (2018). Exploration of rice protein hydrolysates and peptides with special reference to antioxidant potential: Computational derived approaches for bio-activity determination. Trends in Food Science & Technology.

[bb0130] Semyonov D., Ramon O., Shimoni E. (2011). Using ultrasonic vacuum spray dryer to produce highly viable dry probiotics. LWT - Food Science and Technology.

[bb0135] Song H., Marie-Laure V., Dong C.X., Yves L.L., Gwénaël J., Pierre S., Romain J. (2017). Spray drying of probiotics and other food-grade bacteria: A review. Trends in Food Science & Technology.

[bb0140] Sui C., Liang J., Wang F. (2016). Study on direct vat set (DVS) starter culture of *Lactobacillus plantarum* Lp-S2 and its Centrifugation & Freeze-Drying Processes. Food and Machinery.

[bb0145] Sun H., Hua X., Zhang M., Wang Y., Chen Y., Zhang J., Wang Y. (2020). Whey protein concentrate, pullulan, and Trehalose as thermal protective agents for increasing viability of *Lactobacillus plantarum* starter by spray drying. Food Science of Animal Resources.

[bb0150] Wang G., Chen Y., Fei S. (2021). Colonisation with endogenous *Lactobacillus reuteri* R28 and exogenous *Lactobacillus plantarum* AR17-1 and the effects on intestinal inflammation in mice. Food & Function.

[bb0155] Wang J., Zheng S., Feng X., Xia Y., Ai L., Wang G. (2023). Research of carbon source utilization by *Lactiplantibacillus plantarum* AR113. Food and Fermentation Industries.

[bb0160] Wu Y., Zhang X., Cui H., Li H., Hu X. (2023). Isolation, identification and activity analysis of antioxidant peptides from rice wine lees. Journal of Food Measurement and Characterization.

[bb0165] Xiong, T., Liao, L., Huang, T. (2015).Application of spray drying on *Lactobacillus plantarum* NCU116 starter culture.Food and Fermentation Industry, 41(08), 23–29.

[bb0170] Yang J. (2015).

[bb0175] Yao M., Xie J., Du H., McClements D.J., Xiao H., Li L. (2020). Progress in microencapsulation of probiotics: A review. Comprehensive Reviews in Food Science and Food Safety.

[bb0180] Zhang T., Yuan Y., Zhan Y., Cao X., Liu C., Zhang Y., Gai S. (2020). Metabolomics analysis reveals Embden Meyerhof Parnas pathway activation and flavonoids accumulation during dormancy transition in tree peony. BMC Plant Biology.

[bb0185] Zhang X., Pan Y., Zhu H., Wang Z., Zhang X. (2020). Studies on preparation and structural properties of vitamin E nanoemulsion freeze-dried powder. Journal of Chinese Institute of Food Science and Technology.

[bb0190] Zhao X., Gao R., Cui C., Waterhouse D.S. (2024). Structural characteristics, functional properties and in vitro digestibility of rice protein prepared from rice wine lees via an alkaline extraction process or carbohydrate-lipid removal process. Journal of Cereal Science.

[bb0195] Zhao Y., Chen Q., Yue F., Chi X., Yan Y., Jiao W., Cui Z. (2022). Effects of superfine grinding treatment on the properties of mixed powder of grains. China Fruit & Vegetable.

[bb0200] Zhu W., He Q., Gao H., Nitayavardhana S., Khanal S.K., Liu X. (2020). Bioconversion of yellow wine wastes into microbial protein via mixed yeast-fungus cultures. Bioresource Technology.

